# A treatment approach of severe vulvar edema in ovarian hyperstimulation syndrome patient: A case report

**DOI:** 10.18502/ijrm.v21i7.13896

**Published:** 2023-08-23

**Authors:** Leila Sadeghi, Aliyeh Ghasemzadeh, Kobra Hamdi, Nazli Navali, Parvin Hakimi, Laya Farzadi

**Affiliations:** ^1^Department of Obstetrics and Gynecology, Alzahra Hospital, Tabriz University of Medical Sciences, Tabriz, Iran.; ^2^Women's Reproductive Health Research Center, Tabriz University of Medical Sciences, Tabriz, Iran.

**Keywords:** Ovarian hyperstimulation syndrome, Massage, Vulva.

## Abstract

**Background:**

Ovarian hyperstimulation syndrome (OHSS) is a serious life-threatening complication of infertility treatment. Vulvar edema is a disease with various causes and frequent phenomena seen in physiological and pathologic conditions like pregnancy, inflammatory disorders, tumors, idiopathic reasons, and most importantly, in the severe form of OHSS.

**Case Presentation:**

Here, we report a 26-yr-old woman with severe OHSS, recombinant follicle-stimulating hormone therapy. 8 days later, we observed a mild and asymmetrical swelling of the vulva with severe edema in the right labia. Due to the worsening of the vulvar edema even after 15 days of conservative treatment, hand massage and compressive bandaging of the vulva were performed, which caused rapid recovery within 20 min of the case.

**Conclusion:**

Treatment with a hand massage with lubricant gel followed by compressive bandaging resolved the vulvar edema immediately; it is an easy procedure without any adverse events.

## 1. Introduction

Vulvar edema is an uncommon phenomenon associated with various physiological and pathologic conditions, such as inflammatory diseases, vulvovaginal infections, pregnancy, ovarian hyperstimulation, tumors, and iatrogenic causes (1). It may be a local appearance of different systemic conditions, like nephrotic syndrome, congestive heart failure, or inflammatory bowel disease (Crohn's disease) (2). Edema is an excessive liquid accumulation within the interstitial space that occurs following an imbalance in fluid secretion and removal, which finally results in swelling. The predisposing factors for edema included elevation in intravascular hydrostatic pressure, obstruction of lymphatic drainage, an increase in vessel permeability, and a severe decline in plasma oncotic pressure. An interruption and obstruction of inguinal lymph nodes and vessels were observed in feminine genitalia edema (3). Vulvar edema can have a plasmatic origin following the blockage of the vulva's venous drainage. Finally, vulvar edema can lead to some complications like walking difficulties, urination, and examination, and it can also be very painful (4).

Ovarian hyperstimulation syndrome (OHSS) is a potentially serious and iatrogenic complication following ovarian stimulation or ovulation induction. The OHSS incidence is about 2% of assisted conception therapy periods. High-risk women are those whose ovaries are predisposed to an exaggerated response; cases with polycystic ovaries and younger women are also most vulnerable (5). The clinical characteristics of OHSS include enlargement of the ovaries, electrolyte disorders, elevation of coagulability, pleural effusion, and ascites (6). Recommended strategies for declining these risks include low-dose human chorionic gonadotropin (hCG) instead of follicle-stimulating hormone, cycle elimination, embryo cryo-preservation, coasting, cabergoline, and gonadotropin hormone-releasing hormone agonists for ﬁnal follicular maturation instead of hCG (7). Modest vulvar edema has frequently been seen in cases with severe forms of OHSS with signs of abdominal distension and ascites. However, it was reported rarely that cases with massive bilateral vulvar edema which is a distressing condition for both the patients and the gynecologist (8).

In this report, we present a case of massive bilateral vulvar edema following OHSS and intrauterine insemination (IUI). We also review published case reports of vulvar edema following ovulation induction and literature review with any cause of vulvar edema to update the findings.

## 2. Case Presentation

A 26-yr-old female with 2 yr of primary infertility was referred to our tertiary care center as a case of severe OHSS. The participant signed a written informed consent form agreeing to use her medical records for any research purpose. She had undergone IUI 8 days before her presentation at a private institute. It was her first attempt. Her weight and height were 61 kg and 165 cm, respectively. She was treated daily with 150 IU recombinant follicle-stimulating hormone for 4 days.

The IUI process was canceled due to too many mature follicles and gave only the proximity program of coitus interruptus. Luteal support was administrated daily in the form of a 400 mg progesterone vaginal suppository. The participant was admitted due to abdominal distension and also pain. At admission, a gynecological examination of the vulva and vagina revealed mild and asymmetrical swelling of the vulva. On abdominal ultrasound examination, we found enlarged ovaries and severe ascites. Her ovaries at the time of measurement were 17 × 12 cm and 17 × 14 cm. Laboratory tests showed leukocytosis and hypoproteinemia. Levels of serum albumin at the lowest recorded level of proteins were 3.2 g/dL.

There was no hemoconcentration, and the fluid balance was normal. The treatment of this case has consisted of low-molecular-weight heparin, intravenous injection of human albumin, cabergoline, analgesics, and most importantly, maintenance of electrolyte balance. Following observation of the abdominal distension and oliguria, vaginal paracentesis was done by inserting an intra-vaginal catheter through the posterior cul-du-sac on the first day of admission. About 2 L of ascites fluid was drained the first time, and then ascites paracentesis was repeated 5 more times.

**Figure 1:**
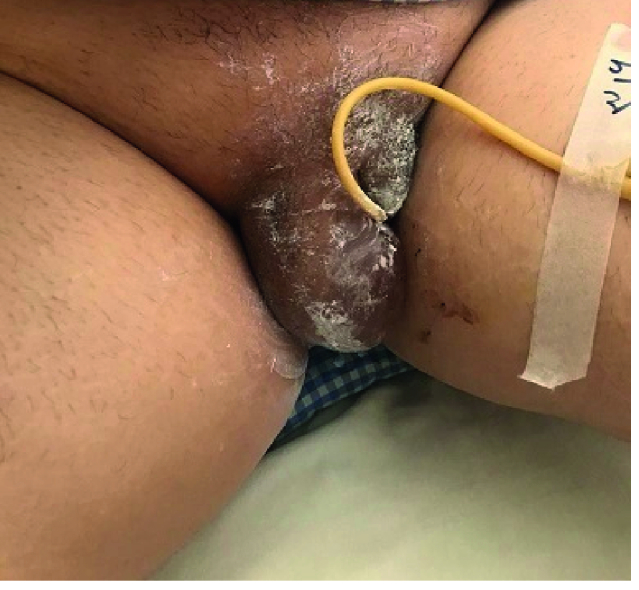
Unilateral vulvar edema 3 day after admission.

**Figure 2:**
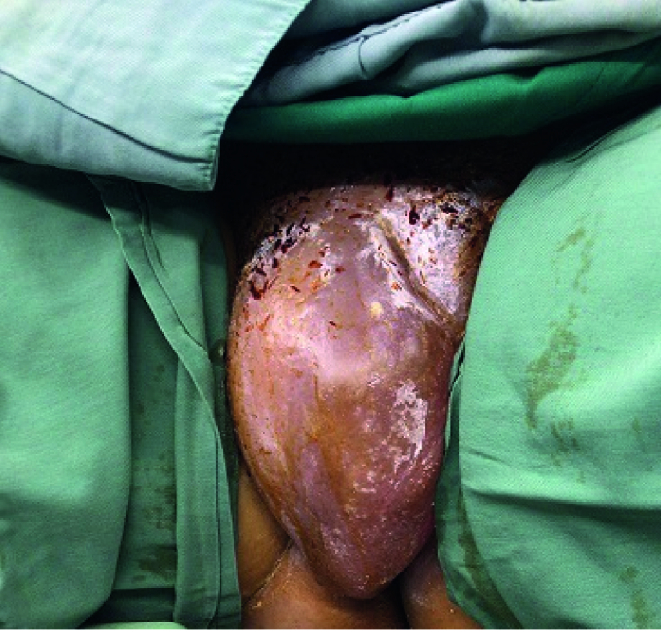
Massive vulva edema related to OHSS before a massage treatment.

3 days after admission, severe asymmetric vulvar edema developed that was painful and prevented her from mobilization accompanied by difficulty passing urine (Figure 1). Then the individual was transferred to the intensive care unit. There was severe edema in the vulva and she had a burning sensation, so a dermatologist was consulted. After rejecting other causes of vulvar edema like trauma, obstruction, infections, and allergies, local treatment of the vulva using ice packs and betamethasone cream daily was proposed. Because of the difficulty in urination, a Foley catheter was carefully inserted. Full expression of vulvar edema was performed on 7 ${}^{\text{th}}$ and 8 ${}^{\text{th}}$ days of her hospitalization and continued for 15 days (Figure 2). A positive serum b-hCG was observed on the 14 ${}^{\text{th}}$ day after IUI.

The edema did not decrease much after releasing more ascites, the trendelenburg position, and topical and conservative treatment. We used massage to treat this participant's vulvar edema. The massage was performed under mild sedation in the operating room because the patient could not tolerate the pain. Hand massage was performed using surgical gloves and lubricant gel. The massage was started using the finger pulp from the inguinal area, and then a gentle and repetitive movement from the valve to the inguinal was performed for 20 min. Vulvar edema resolved ultimately. Then a pressure dressing was applied with compressive bandaging to prevent the edema from returning.

**Figure 3:**
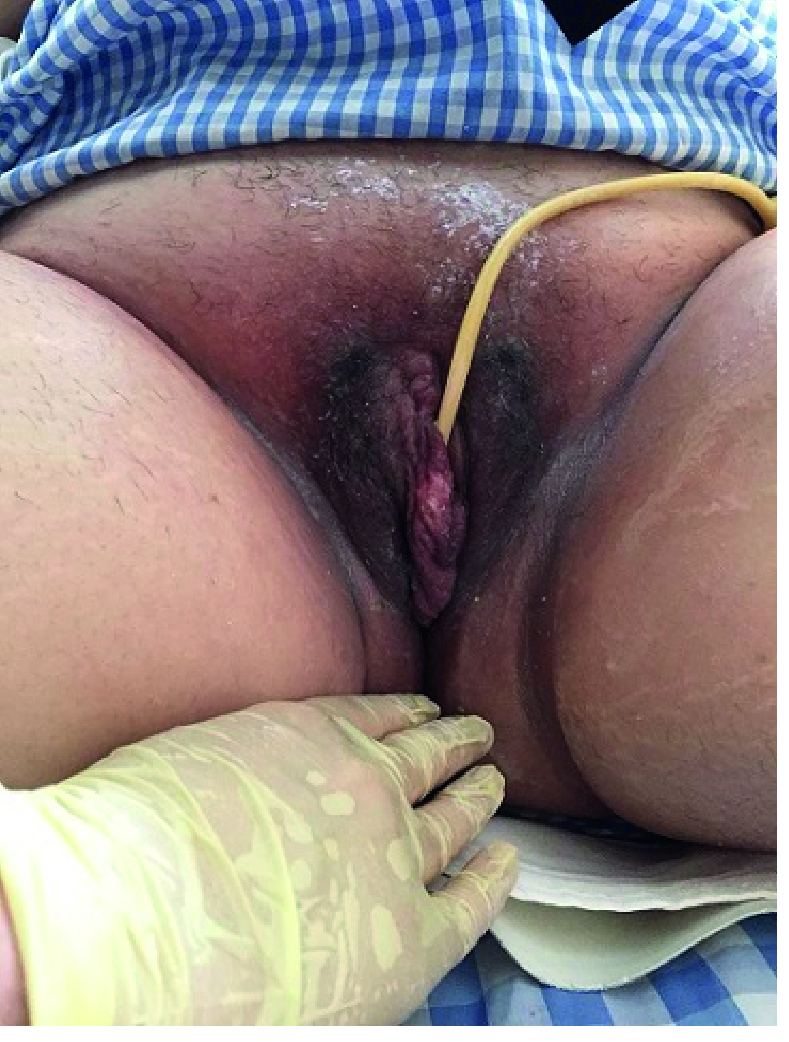
Resolved completely OHSS-induced severe vulva edema after 20 min of massage followed by compressive bandaging.

Before compressive bandaging, we placed a surgical sponge on the genital area to protect the vulva and also avoid skin damage following contact with the bandage. 8 hr later, we opened the bandage, the vulvar area was normal, and the patient did not feel any pain and was relieved (Figure 3). The patient was asymptomatic at the time of her first obstetrical visit and was discharged home on the 17 ${}^{\text{th}}$ day after admission. She spontaneously aborted a blighted ovum at 8 wk of gestation spontaneously. She became pregnant again 2 months after the abortion. At the time of writing this paper, the patient was 27 wk pregnant.

### Ethical considerations

A written informed consent was obtained from patient to use her medical records for any research purpose.

## 3. Discussion

In this case study, we used cold therapy with an ice pack and antibiotics to initiate the cure period. We found that hand massage and compressive bandages have a more effective impact on resolving vulvar edema. This method was an easy procedure without any adverse events.

Severe vulvar edema is an uncommon pathological condition in pregnancy. Labial edema can result from different clinical conditions (8). OHSS is a scarce phenomenon, and very few publications on OHSS involve progressive vulva edema development. An association between massive vulvar edema and OHSS was described. In the previous study, conservative management with heparin, human albumin, and lactated Ringer's was done. They used local application of cortisone, useful antibiotic ointment twice daily, and ice packs for treatment of vulvar edema, which after 1 wk backed to the normal vulva (9).

Another study reported a case study of severe OHSS with ascites, abdominal distension, and severe unilateral vulvar edema following the attempt to become pregnant with in vitro fertilization. A blood test showed a significant decline in the p-albumin level, which led to the infusion of human albumin daily for 1 wk. Edema was resolved 1 wk after the treatment, giving the impression that hypoalbuminemia is one of the crucial factors in the development of vulvar edema (10). A study described a rare case of unilateral vulvar edema in a 23-yr-old woman who showed OHSS due to an occult inguinal hernia. A concern for skin necrosis and skin integrity breakage was reported (11).

After diagnosis, to resolve the problem, the patient was placed on bed rest with a slight elevation of the hip. This strategy helps to avoid further accumulation of ascites underneath the labia majora. In a rare case (12), stated bilateral vulvar edema followed the OHSS because the patients potentially had a patent canal of Nuck. The treatment procedure demonstrated in previous studies, which used the ice pack, hypertonic saline bags, bed rest, diuretic administration, local and oral antibiotics, Trendelenburg position, painkillers, lactated Ringer's solution, insertion of a Foley's catheter, low-molecular-weighted heparin, human albumin; and elastic stockings to prevent venous thrombosis, mechanical drainage via a small incision on the labia to allow fluid flow, manual drainage, multilayer compression therapy, and multichambered pneumatic compression device and skincare (1). The average time to resolve the edema is in the range of 7-14 days (1). In the case report with severe vulvar edema post OHSS, local myrrh and an ice pack were used for patient management. Surprisingly, following this treatment strategy by using myrrh, edema improved after 2 days. Myrrh is composed of sesquiterpenes, water-soluble gum, and unstable essential oils (13).

In another case of post-paracentesis vulvar edema in an OHSS patient, the leading cause of bilateral vulvar edema was a fistulous tract created (iatrogenically) among the subcutaneous tissues and peritoneal cavity. The patient received the local treatment of an ice pack, hydrocortisone, Gentamycin ointment alternatively, and intravesical albumin. A progressive reduction in symptoms was observed, and edema resolved entirely in 4 days (14). Besides, a case series was conducted to treat vulvar edema by manual lymphatic drainage and multilayer compression therapy in 2015. Total improvement of the edema happened in 2-5 days of treatment (1). Recently, in a rare case series study for the treatment of perineal post-related vulvar edema and necrosis, they used local dressings using glycerin and magnesium sulfate to reduce the tissue edema (15).

Presenting gross vulvar edema along with preeclampsia is not a common phenomenon, and its significance does not establish entirely. A 4-case series of this disorder was evaluated in the study tried by Abdul et al. (16). 3 patients showed the severity of disease characterized by renal failure with oliguria, adverse peritoneal outcome, hemolysis, elevated liver enzymes and low platelets syndrome, and intrauterine growth restriction. It suggested that the presence of gross vulvar edema in cases with preeclampsia may be an important indicator of disease severity and, most prominently, a biomarker of deteriorating fetomaternal condition (16).

One study reported a 15-yr-old primigravida case with spontaneous massive vulvar edema following tocolysis during the 33 wk of gestation. Antibiotics, corticoids, and analgesics as pain relief were used for the prevention of vulvar edema progression. During the 10 days of this treatment strategy, the vulvar edema improved, and the patient delivered a live newborn by spontaneous vaginal delivery with a standard postpartum period. This study emphasized the importance of conservative attempts for treatment of the vulvar edema complicating tocolysis (17).

Similarly, another case report of spontaneous massive vulva edema during the pregnancy was reported by a previous study on a 24-yr-old primigravida during the 28 wk of gestation, who had a twin, with one anencephaly and polyhydramnios. After an emergency cesarean section and within 12 hr post-operatively, the vulvar edema subsided. This spontaneous resolution of the edema after delivery has been reported in several studies (16).

##  Conflict of Interest

The authors declare that there is no conflict of interest.

## References

[B1] Pinto de Silva MP, Bassani MA, Miquelutti MA, Andrade Marques Ad, do Amaral MTP, de Oliveira MMF, et al (2015). Manual lymphatic drainage and multilayer compression therapy for vulvar edema: A case series. Physiother Theory Pract.

[B2] Hadzavdic SL, Jovic A, Hadzavdic A, Grgec DL (2018). Vulvar oedema. Contact Dermatitis.

[B3] Trayes KP, Studdiford JS, Pickle S, Tully AS (2013). Edema: Diagnosis and management. Am Fam Physician.

[B4] Kiyama M, Kanayama K, Roh S, Hattori Y, Oba J, Iida T, et al (2020). Multiple symmetric lipomatosis with vulvar involvement: A rare case report associated with walking difficulty and urination disorder. Eur J Plast Surg.

[B5] Nelson SM

[B6] Ma XW, Yin JW, Yang R, Yang S, Li J, Wang Y, et al (2022). [Clinical characteristics of severe late-onset ovarian hyperstimulation syndrome and its impact on the live birth outcome of IVF-ET]. Zhonghua Fu Chan Ke Za Zhi.

[B7] Leitao VMS, Moroni RM, Seko LMD, Nastri CO, Martins WP (2014). Cabergoline for the prevention of ovarian hyperstimulation syndrome: Systematic review and meta-analysis of randomized controlled trials. Fertil Steril.

[B8] Kovač V, Reljič M, Bizjak T (2019). Causes of massive vulvar edema in patients with severe ovarian hyperstimulation syndrome: A case report and literature review. Am J Case Rep.

[B9] Coccia ME, Bracco GL, Cattaneo A, Scarselli G (1995). Massive vulvar edema in ovarian hyperstimulation syndrome. A case report J Reprod Med.

[B10] Sopa N, Toftager M (2018). Post laparoscopic massive vulvar edema in woman with ovarian hyperstimulation syndrome. Int J Case Rep Images.

[B11] Kalafat E, Acar D, Aytac R (2016). Swollen labia majora: An unusual presentation of occult inguinal hernia secondary to ovarian hyperstimulation syndrome. Taiwan J Obstet Gynecol.

[B12] Cline DL (1996). Massive vulvar edema in ovarian hyperstimulation syndrome. J Reprod Med.

[B13] Hijazi A, Al-Jaroudi D (2017). Myrrh for treatment of severe vulvar edema in ovarian hyperstimulation syndrome. Case Rep Women's Health.

[B14] Bhairavi S, Rajendran J, Dash S, Dash S (2016). Postparacentesis vulvar edema in ovarian hyper stimulation syndrome. Int J Reprod Contracept Obstet Gynecol.

[B15] Habek D, Habek JČ, Vujic B (2005). Nontraumatic chyloperitoneum in pregnancy. Eur J Obstet Gynecol Reprod Biol.

[B16] Abdul MA, Odogwu K, Madugu N (2011). Gross vulva odema complicating severe pre-eclampsia/eclampsia: A case series. Niger J Med.

[B17] Mulisya O, Mastaki M, Gertrude T, Tasi K, Mathe JK (2018). Spontaneous massive vulvar edema in pregnancy: A case report. Case Rep Obstet Gynecol.

